# Effects of the Long-term Care Insurance on Health Among Older Adults: A Panel Data From China

**DOI:** 10.34172/ijhpm.2023.7664

**Published:** 2023-08-22

**Authors:** Xin Ye, Mingzheng Hu, Hugo Lin

**Affiliations:** ^1^Institute for Global Public Policy, Fudan University, Shanghai, China; ^2^LSE-Fudan Research Centre for Global Public Policy, Fudan University, Shanghai, China; ^3^School of Public Health, Peking University, Beijing, China; ^4^China Center for Health Development Studies, Peking University, Beijing, China; ^5^CentraleSupélec, Paris-Saclay University, Paris, France

**Keywords:** Long-term Care Insurance, Health, Older Adults, China

## Abstract

**Background:** China’s long-term care insurance (LTCI) has been launched since 2016 to ensure that older disabled people obtain affordable care services. However, rigorous evaluations of the health effects of China’s LTCI pilots have been limited. This paper aimed to examine the effects of LTCI on health among older adults aged 60 years and above.

**Methods:** Drawing from panel data of the China Health and Retirement Longitudinal Study (CHARLS), we used a propensity score matching (PSM) and difference-in-difference (DID) approach to identify the health effects of the LTCI program and reduce the selection bias. Further, heterogeneity of the effects was examined by physical and intellectual function to evaluate whether the effects differed among subgroups of older population.

**Results:** The implementation of LTCI significantly improved self-rated health (β = 0.15, *P*<.05) and cognitive function (β = 0.59, *P*<.01) for older adults. The results were robust when keeping only those living in pilot cities (β = 0.31, *P*<.05 for self-rated health status; β = 0.98, *P*<.001 for cognitive function) or non-pilot cities (β = 0.14, *P*<.05 for self-rated health status; β = 0.60, *P*<.01 for cognitive function) as the control group. The effects of LTCI were especially manifested in older adults with physical disability (β = 0.13, *P*<.01 for self-rated health; β = 0.76, *P*<.001 for cognitive function) or intellectual disability (β = 0.16, *P*<.01 for self-rated health).

**Conclusion:** From a policy perspective, these findings suggested that LTCI in China could benefit the health outcomes of older adults, especially those with physical or cognitive disabilities. Policy-makers can target resources more effectively to improve health outcomes for the most vulnerable populations.

## Background

**Key Messages**

**Implications for policy makers**

- The implementation of long-term care insurance (LTCI) significantly improved self-rated health and cognitive function for older adults.
- The effects of LTCI were especially manifested in older adults with physical disability or intellectual disability.
- Our study has important implications for the development of LTCI in China and other middle-income or developing countries, which are facing rapid population aging and increasing demands for LTC.

**Implications for the public**

China’s rapidly aging population has created a growing demand for long-term care insurance (LTCI). The implementation of LTCI in China had positive effects on the health of older adults. Specifically, LTCI improved self-rated health and cognitive function in this group, and the effects were especially noticeable for older adults with physical or intellectual disabilities. The implications for the public were significant, as they suggested that investing in LTCI programs could promote the health and well-being of older adults who were most vulnerable. It also highlighted the importance of developing policies that addressed the long-term care (LTC) needs of older adults, so as to secure their health.

China’s rapidly aging population has created a growing demand for the long-term care (LTC) services.^1^ In China, the number of people aged 60 and above has been 267 million by 2021 and it is expected to reach 480 million by 2050.^2^ Informal care provided by family members has long been the main source of care for older adults.^3^ However, as a result of reduced family size, large-scale domestic migration and a decline in cohabiting with older adults, fewer people are taking on informal care in households and the risk for unmet LTC needs is growing substantially in China.^4,5^ About one-third of functionally disabled people age 45 and older who need help with one or more activities of daily living (ADLs) or instrumental ADLs have their needs unmet.^6^ Hence, the demands for formal LTC outside traditional informal networks are increasing.^7^

 To ensure that older disabled people receive affordable care services and alleviate the care burdens on family members, the Chinese government launched a long-term care insurance (LTCI) policy experimentation in 15 cities and 2 provinces in 2016.^8^ In the pilot phase, LTCI was mainly financed by existing public health insurance schemes to cover basic care costs for daily living and related medical expenses for eligible beneficiaries with long-term disabilities.^7^ LTC services can be broadly divided into three types, eg, home care, institutional care and hospital care. The types and frequencies of LTC services available to beneficiaries depends on the severity of their disability. Reimbursement rates vary depending on the type of LTC services provided and are usually higher for home care.^7^ Theoretically, an increase in formal LTC utilization could help maintain or improve the health status of disabled older adults, who are the primary recipients of LTCI benefits. More details of LTCI can be seen in Text S1 and Table S1 (Supplementary file 1).

 Most studies have examined the health effects of LTCI in developed countries. Previous studies assessing the effects of LTCI or different types of formal care on health outcomes of older adults produced mixed results. For example, an increase in public home care program generosity was associated with an improvement in self-reported health in Canada.^9^ In the context of Korea, the LTCI policy was associated with lower mortality risk among care beneficiaries,^10,11^ and their cognitive function and disability declined less.^12^ Yet another study found that subsidies for LTC had no effects on mortality.^13^ Several studies showed that LTCI had little effects on health outcomes of frail older adults,^14-16^ or the decline in formal home healthcare had no adverse health effects,^17^ but reduced independent living among older adults in the United States.^18^ In the coming decades, developing countries with different social systems, cultures and socio-economic environments will be the main force of global population ageing.^19^ Therefore, it is necessary to examine the health effects of LTCI in developing countries like China.

 Up to now, rigorous evaluations of the health effects of China’s LTCI pilots have been limited. Available evidence showed that the introduction of LTCI led to an improvement in overall health^20,21^ and quality of life,^22^ including self-reported health,^7,23^ the ADL,^23^ mental health,^23^ cognitive function,^24^ and a reduction in one-year mortality risk.^7^ However, the potential effects and heterogeneity of LTCI on comprehensive health outcomes of older adults are still unclear. Understanding the health impacts of LTCI is important given the aging population in China and the need for effective policies to support older adults’ health and well-being. This study is, to the best of our knowledge, one of the first to examine the health effects of China’s first pilots of the public LTCI program on older adults aged 60+, utilizing a quasi-experimental design of the LTCI policy. We included 15 pilot cities and also two provinces (ie, Jilin and Shandong provinces) as the study frame, which has never been done before. Our goal is to conduct a comprehensive health assessment of the LTCI policy and provide policy relevance to other middle-income or developing countries.

## Methods

###  Data and Sample

 We used data from the 2015 and 2018 waves of the China Health and Retirement Longitudinal Study (CHARLS), which covered the periods before and after the introduction of LTCI. CHARLS is a nationally representative longitudinal survey.^25^ So far, a total of four waves of surveys have been completed in 2011, 2013, 2015, and 2018, respectively. We targeted respondents aged 60+ years, who were the most relevant group to the LTCI policy. A total of 13 730 respondents aged 60+ were interviewed in the 2015 survey, of whom 9087 (66.18%) were interviewed again in 2018. 4643 (33.82%) respondents died or were lost to follow-up prior to the 2018 survey, and 47 respondents were dropped due to missing values in control variables. Our main study sample is a panel composed of 9040 older adults from 122 cities. Based on the pre-post treatment-control design, we included health insurance enrollees who were covered by the local LTCI pilot between 2015 and 2018 into the treatment group (N = 314). The remaining were assigned to the control group (N = 8726).

 Panel attrition may be a source of bias if respondents were lost to follow-up non-randomly and their sample attrition was systematically related to the treatment variable (ie, the LTCI coverage) conditional on the characteristics we controlled for in equation. The fixed-effects specification in our main analysis mitigated the concern regarding attrition due to time-invariant sources. To further examine whether the nonresponse was associated with the treatment, we used the full sample of CHARLS 2015 and constructed an indicator of whether the respondent was lost to follow-up in 2018. We then regressed this indicator on individual treatment status and a set of demographic and socioeconomic variables in 2015. In Table S2, the coefficients of the treatment variable were statistically insignificant, suggesting that the possibility of respondents lost to follow-up in 2018 was independent with the LTCI coverage, and that potential attrition bias was unlikely to be a concern.

###  Outcome Variables

 We used multiple measures of health as outcome variables in CHARLS, including self-reported health, physical function, kinds of chronic diseases, cognitive function, and depression.

 Self-reported health was measured according to a five-category survey question: “How do you rate your health at present?” We constructed a continuous indicator with a value of 0 if the respondent reported “poor” health, a value of 1 for “fair” health, and a value of 2 for “good” health.

 ADLs based on the Barthel Index was used to access physical function: feeding, bathing, grooming, dressing, bowels, bladder, toilet use, transfers (bed to chair and back), mobility (on level surface), and stairs. Although the assessment of ADLs in CHARLS did not use Barthel Index, ADLs in CHARLS were converted to conform to the measurement of Barthel Index following previous literature (Table S3).^8^ The total score ranged from 0–100, with a higher score indicating a greater independence. 0–60 meant moderate or severe disability, and 61–100 meant mild disability or full independence.^8^

 Kinds of chronic diseases were calculated according to respondents’ answers to the question of whether they had at least one chronic disease diagnosed by a doctor, including hypertension, dyslipidemia, diabetes, cancer, chronic lung diseases, liver diseases, heart diseases, stroke, kidney diseases, digestive system diseases, arthritis, or asthma.

 Cognitive function measured respondents’ abilities of orientation, attention, episodic memory and mental intactness, which was presented as the total score in the following four aspects: (*a*) naming the day, week, month, year, and the season (0 to 5 scores); (*b*) subtracting a number by five times (0 to 5 scores); (*c*) drawing a picture on the paper (0 to 1 score); (*d*) memorizing and recalling ten words (average 0 to 10 scores). The range was 0-21, and higher score meant better cognitive function.^26^ A score of 6 or less was used as a cut point for having intellectual disability.^27^

 Depression was detected by using the 10-item Center for the Epidemiological Studies of Depression Short Form (CES-D-10).^28^ The subjects rated how often each emotion had occurred in the past week on a 4-point scale, ranging from 0 (“none”) to 3 (“most of the time”). A total score of CES-D-10 ranged from 0 to 30.

###  Independent Variables

 We constructed an indicator of whether the respondent was covered by LTCI. LTCI coverage was based on the pilot timing at the city level and local requirements for public health insurance status (ie, Urban Employee Basic Medical Insurance, Urban Resident Basic Medical Insurance, and Urban and Rural Resident Basic Medical Insurance). In our sample, 15 cities had implemented the program by the 2018 survey, including 11 cities in the 2016 officially announced pilot list and 4 cities in which the local government launched the program during 2015-2018. All 15 pilot cities offered LTCI coverage for Urban Employee Basic Medical Insurance enrollees. 2 cities also covered urban and rural residents enrolled in Urban and Rural Resident Basic Medical Insurance.

 In the 2018 survey, 314 respondents were living in pilot cities and covered by the local LTCI, who were classified as the treated group. The remaining 8726 respondents uncovered by LTCI during 2015-2018 were classified as the control group, no matter whether they lived in pilot cities or not. That is, the specific groups of people included in the control group were (1) those uncovered by the local LTCI and living in pilot cities; (2) those uncovered by the local LTCI and living in non-pilot cities.

###  Covariates

 Covariates included age (continuous), gender (male/female), marital status (single/married), education level (illiterate/literate/primary school/junior high school and above), residence (urban/rural), smoking (no/yes or ever), drinking (no/yes or ever), and the number of living children (continuous).

###  Statistical Analysis

 To evaluate the health effects of LTCI and to avoid the confounding health effects of insurance coverage other than LTCI, we applied a difference-in-difference (DID) strategy with individual fixed effects (FE) to the two-year panel data: 2015 before LTCI and 2018 after LTCI. The DID approach compared the changes in health outcomes over time between those who had participated in the LTCI program (the treatment group) and those who had not (the control group) while controlling for other factors that might affect health outcomes. By comparing changes in health outcomes before and after the implementation of the LTCI program in the treatment group with the control group, the study could isolate the effects of LTCI coverage from other factors. DID is a well-established method in health policy research and has been shown to be effective in evaluating the impact of policy interventions. The following equation is estimated:

*y*_ict_= *β*_1_*Treat*_ic_ × *Post*_t_+ *β*_2_*X*_ict_+ *τ*_t_+ *a*_i_+ *ε*_ict_

 where *y*_ict_ denotes the health outcomes of individual *i* living in city *c* in year *t*. In the independent variable *Treat*_ic_ × *Post*_t_, *Post*_t_ is a dichotomous variable that is 0 for 2015 and 1 for 2018, and *Treat*_ic_ is a dummy variable for individual treatment status. *X*_ict_ is a vector of individual time-varying characteristics that includes the respondent’s age, marital status, residence, smoking, drinking, and the number of living children. *τ*_t_ is year FE. *a*_i_ is individual FE that account for all time-invariant factors that may affect the outcome variables. *ε*_ict_ is a random error term. Standard errors are clustered at the city level to account for possible correlation in outcomes between older adults in the same city.

 The key assumption of the DID specification is that the potential trends in the outcome variables in both the treated and control groups should be parallel without the implementation of LTCI. We tested the assumption by using the 2011–2018 CHARLS panel and found no evidence of the differential pre-trends (Table S4). On the other hand, due to the large difference in sample size between the treatment group and the control group, the conclusions may be influenced by selection bias due to sample mismatch between the treatment and control groups.^29,30^ We combined the DID regression and the propensity score matching (PSM) method to achieve “double robustness.”^31^ We first estimated a logit model to obtain the propensity score—that is, the probability of being in the treatment group given the set of baseline observable characteristics. With the estimated propensity score, we performed the DID analyses in the common support, so that the probability of LTCI coverage was similar between two groups. We used the nearest neighbor matching, after which all the observable characteristics were well balanced and propensity score distributions were more similar between the two groups, which further tested the validity and reliability of the results and proved that the sample equilibrium hypothesis was satisfied (Figure S1).

 In addition, there could exist the “spillover effect” of LTCI, which refers to the potential beneficial impact of the program on those who are not direct beneficiaries of the program but are covered by it. In our study, the “spillover effect” could be manifested as improvements in health outcomes among non-LTCI beneficiaries residing in regions with high coverage rates of LTCI (Chongqing, Guangdong, Heilongjiang, Hubei, Jiangsu, Jilin, Shandong, Shanghai, and Zhejiang). The distribution of treated and control groups in the spillover study is presented in Table S5. We also employed panel data analysis and the DID approach to compare the changes in health outcomes between areas with high and low LTCI coverage rates over time. It was found in Table S6 that individuals living in areas with high LTCI coverage rates did not manifest better self-rated health or cognitive function than those living in areas with low coverage rates, even after controlling for other variables such as age, sex, marital status, education level, etc, indicating that the spillover benefits of LTCI could be small.

 Further, the robustness test was carried out by selecting a different control group. This was done to ensure that the results were not affected by the selection of the control group and were reliable. The robustness test included two control groups - one comprising older adults living in pilot cities who were not covered by the LTCI program during the study period, and the other comprising older adults living in non-pilot cities who were not covered by the LTCI program during the study period. The process involved selecting the two new control groups, and then performing the same statistical analysis as done for the original control group. Results obtained from the original control group (uncovered respondents during 2015-2018) were compared with those obtained from the two new control groups. Lastly, we examined heterogeneity of health effects across different populations, ie, older adults with/without physical disability and older adults with/without intellectual disability, so as to provide information on how to target resources and interventions more effectively to improve health outcomes for the most vulnerable populations.

## Results

Table 1 presents descriptive statistics for the treated and control groups in wave 2015 and 2018. T-test was applied for continuous variables and analysis of variance (ANOVA) for category variables, indicating the significance level of pairwise comparisons prior to and after the LTCI pilot. The treatment group was statistically similar to the control group in kinds of chronic diseases (*P* = .12) and age (*P* = .24). The treatment group were more likely to be female (*P* < .001), single (*P* = .001), educated (*P* < .001), from rural areas (*P* < .001), smoking (*P* = .004), drinking (*P* = .019), and had fewer living children (*P* < .001). They had better self-rated health, worse physical function, better cognitive function, and less depression (*P* < .001).

**Table 1:** Summary Statistics (2015-2018 Panel)

	**Wave 2015 (n = 9040)**			**Wave 2018 (n = 9040)**		
	**Treated (n = 314)**	**Control (n = 8726)**	***P***	**Treated (n = 314)**	**Control (n = 8726)**	***P***
**Outcome Variables**						
Self-rated health status (0-2)	1.12 (0.04)	0.98 (0.01)	<.001	1.09 (0.04)	0.87 (0.01)	<.001
Physical function (0-100)	57.10 (1.98)	67.83 (0.33)	<.001	62.60 (1.89)	69.66 (0.31)	<.001
Kinds of chronic diseases (0-12)	2.43 (0.11)	2.27 (0.02)	.12	0.71 (0.05)	0.76 (0.01)	.38
Cognitive function (0-21)	12.13 (0.22)	9.03 (0.05)	<.001	11.30 (0.23)	8.19 (0.05)	<.001
Depression (0-30)	5.78 (0.34)	8.39 (0.07)	<.001	5.69 (0.34)	7.79 (0.07)	<.001
**Covariates**						
Age	68.51 (0.40)	68.06 (0.07)	.24	71.51 (0.40)	71.06 (0.07)	.24
Gender			<.001			<.001
Male	127 (40.45%)	4552 (52.17%)		127 (40.45%)	4552 (52.17%)	
Female	187 (59.55%)	4174 (47.83%)		187 (59.55%)	4174 (47.83%)	
Marital status			.001			.001
Single	40 (12.74%)	1788 (20.49%)		53 (16.88%)	2181 (24.99%)	
Married	274 (87.26%)	6938 (79.51%)		261 (83.12%)	6545 (75.01%)	
Education level			<.001			<.001
Illiterate	25 (7.96%)	3056 (35.02%)		25 (7.96%)	3056 (35.02%)	
Literate	36 (11.47%)	1809 (20.73%)		36 (11.47%)	1809 (20.73%)	
Primary school	83 (26.43%)	2090 (23.95%)		83 (26.43%)	2090 (23.95%)	
Junior high school and above	170 (54.14%)	1771 (20.30%)		170 (54.14%)	1771 (20.30%)	
Residence			<.001			<.001
Urban	64 (20.38%)	2054 (23.54%)		65 (20.70%)	2096 (24.02%)	
Rural	250 (79.62%)	6672 (76.46%)		249 (79.30%)	6630 (75.98%)	
Smoking			.004			.002
Never	145 (46.18%)	4764 (54.60%)		146 (46.50%)	4832 (55.37%)	
Yes or ever	169 (53.82%)	3962 (45.40%)		168 (53.50%)	3894 (44.63%)	
Drinking			.019			.014
Never	148 (47.13%)	4714 (54.02%)		145 (46.18%)	4643 (53.21%)	
Yes or ever	166 (52.87%)	4012 (45.98%)		169 (53.82%)	4083 (46.79%)	
Number of living children	2.43 (0.08)	3.25 (0.02)	<.001	2.36 (0.08)	3.08 (0.02)	<.001

Note: Standard deviations are in parentheses; standard errors clustered at the city level are in brackets in the last column.

Table 2 reports the estimated effects of LTCI on health outcomes, including self-rated health status, physical function, kinds of chronic diseases, cognitive function, and depression. Column 1 in Table 2 demonstrates results from the DID estimate that LTCI coverage might improve health outcomes, but marginally non-significant. In column 2, using the PSM matched sample, the DID estimates for self-reported health (β = 0.15, *P* < .05) and cognitive function (β = 0.59, *P* < .01) were significantly positive. For physical function (β = 1.77, *P* > .05), kinds of chronic diseases (β = -0.14, *P* > .05), and depression (β = -0.41, *P* > .05), there were no significant improvements.

**Table 2:** Effects of Long-term Care Insurance on Health Outcomes

**Dependent Variables**	**Coefficient on Treat × Post**	
	**DID**	**DID With Matching**
Self-rated health status	0.09 (0.04)	0.15* (0.04)
Physical function	3.38 (2.46)	1.77 (3.61)
Kinds of chronic diseases	-0.13 (0.09)	-0.14 (0.19)
Cognitive function	0.10 (0.27)	0.59** (0.20)
Depression	-0.31 (0.08)	-0.41 (0.37)
Year FE	Y	Y
Individual FE	Y	Y

Abbreviations: DID, difference-in-difference; FE, fixed effects; Y, yes. Note: Standard errors are clustered at the city level. The significance levels of 0.1%, 5%, and 1% are denoted by ***, **, and *, respectively. All regressions control for year FE, individual FE, and individual covariates.

 As a robustness check, we kept only the uncovered older adults living in pilot cities (N = 1450) or the uncovered sample living in non-pilot cities (N = 7276) as the control group. This was done to ensure that the results were not affected by the selection of the control group and were reliable. In Table 3, we found that the results did not change much. In columns 2, LTCI significantly improved self-rated health status (β = 0.31, *P* < .05), reduced kinds of chronic diseases (β = -0.42, *P* < .001), improved cognitive function (β = 0.98, *P* < .001), and alleviated depression (β = -1.49, *P* < .001) compared to uncovered older adults living in pilot cities. In columns 4, LTCI significantly improved self-rated health status (β = 0.14, *P* < .05) and cognitive function (β = 0.60, *P* < .01) compared to uncovered older adults living in non-pilot cities.

**Table 3:** Effects of Long-term Care Insurance on Health Outcomes (Control Groups Limited to Uncovered Older Adults Living in Pilot Cities or Those Living in Non-pilot Cities)

**Dependent Variables**	**Control Groups Limited to Uncovered Older Adults Living in Pilot Cities (N = 1450)**		**Control Groups Limited to Uncovered Older Adults Living in Non-pilot Cities (N = 7276)**	
	**DID**	**DID With Matching**	**DID**	**DID With Matching**
Self-rated health status	0.05 (0.04)	0.31* (0.11)	0.10* (0.04)	0.14* (0.07)
Physical function	4.02 (2.51)	5.07 (4.37)	3.26 (2.51)	2.23 (3.67)
Kinds of chronic diseases	-0.12 (0.10)	-0.42*** (0.05)	-0.13 (0.09)	-0.15 (0.19)
Cognitive function	0.34 (0.26)	0.98*** (0.21)	0.05 (0.27)	0.60** (0.21)
Depression	-0.20* (0.10)	-1.49*** (0.44)	-0.10 (0.09)	-0.80 (0.57)
Year FE	Y	Y	Y	Y
Individual FE	Y	Y	Y	Y

Abbreviations: DID, difference-in-difference; FE, fixed effects; Y, yes. Note: Standard errors are clustered at the city level. The significance levels of 0.1%, 5%, and 1% are denoted by ***, **, and *, respectively. All regressions control for year FE, individual FE, and individual covariates.

 Older adults covered by LTCI became eligible for service benefits when they had moderate or severe physical disability. We provided indirect evidence by further investigating whether the effects of LTCI varied across older adults with or without physical disability in Table 4. In our sample, 25.85% had moderate or severe physical disability in pretreatment year 2015. LTCI significantly improved their self-rated health status (β = 0.13, *P* < .01) and cognitive function (β = 0.76, *P* < .001). Table S7 added an interaction term between LTCI coverage and physical disability in pretreatment year 2015 using the same dataset. It was used to examine whether there existed heterogeneous effects of LTCI by physical function. The effects of LTCI on cognitive function (β = 2.75, *P* < .01) varied significantly between older adults with or without moderate or severe physical disability.

**Table 4:** Effects of Long-term Care Insurance on Health Outcomes, by Physical Function

**Dependent Variables**	**Moderate or Severe Physical Disability** **(N = 2204)**		**Mild Physical Disability or Full Independence** **(N = 6323)**	
	**DID**	**DID With Matching**	**DID**	**DID With Matching**
Self-rated health status	0.07 (0.05)	0.13** (0.04)	0.46* (0.20)	0.39 (0.21)
Physical function	-0.10 (0.10)	-0.10 (0.10)	-0.50 (0.38)	-1.11 (1.27)
Kinds of chronic diseases	0.05 (0.27)	0.76*** (0.19)	1.12 (0.88)	2.34 (1.83)
Cognitive function	-0.13 (0.09)	-0.57 (0.43)	-0.16 (0.40)	-1.21 (2.45)
Depression	-0.20* (0.10)	-1.49*** (0.44)	-0.10 (0.09)	-0.80 (0.57)
Year FE	Y	Y	Y	Y
Individual FE	Y	Y	Y	Y

Abbreviations: DID, difference-in-difference; FE, fixed effects; Y, yes. Note: Standard errors are clustered at the city level. The significance levels of 0.1%, 5%, and 1% are denoted by ***, **, and *, respectively. All regressions control for year FE, individual FE, and individual covariates.

 Intellectual disability is one of the inclusion criteria explicitly specified in the LTCI eligibility criteria in some cities (eg, Qingdao of Shandong province and Guangzhou of Guangdong province). In addition, intellectual disability can also be regarded as a kind of disability. We therefore investigated whether the effects of LTCI varied across older adults with or without intellectual disability (cut-off point at 6) in Table 5. In our sample, 28.58% had intellectual disability in pretreatment year 2015. For older adults with intellectual disability, LTCI significantly improved self-rated health status (β = 0.20, *P* < .01). For older adults without intellectual disability, LTCI had no effects on health outcomes. Table S8 added an interaction term between LTCI coverage and intellectual disability in pretreatment year 2015. The effects of LTCI on self-rated health (β = -0.24, *P* < .05) and physical function (β = 10.51, *P* < .05) varied with intellectual status.

**Table 5:** Effects of Long-term Care Insurance on Health Outcomes, by Intellectual Function

**Dependent Variables**	**Intellectual Disability** **(N = 2329)**		**No Intellectual Disability** **(N = 5820)**	
	**DID**	**DID With Matching**	**DID**	**DID With Matching**
Self-rated health status	0.16** (0.06)	0.20** (0.07)	0.06 (0.05)	0.09 (0.09)
Physical function	0.66 (2.72)	2.79 (4.04)	3.38 (3.52)	0.65 (2.06)
Kinds of chronic diseases	-0.10 (0.16)	-0.34 (0.26)	-0.16 (0.12)	-0.14 (0.19)
Cognitive function	-0.24 (0.15)	-1.07 (0.74)	-0.34 (0.11)	-0.37 (0.61)
Depression	-0.20* (0.10)	-1.49*** (0.44)	-0.10 (0.09)	-0.80 (0.57)
Year FE	Y	Y	Y	Y
Individual FE	Y	Y	Y	Y

Abbreviations: DID, difference-in-difference; FE, fixed effects; Y, yes. Note: Standard errors are clustered at the city level. The significance levels of 0.1%, 5%, and 1% are denoted by ***, **, and *, respectively. All regressions control for year FE, individual FE, and individual covariates.

## Discussion

 With the rapid aging of the population and the weakening of traditional family care, the demand for formal LTC services among China’s elderly population is escalating. To explore a systematic financing mechanism, the government officially launched LTCI pilots in 2016. However, despite the rapid expansion of LTCI in China, the effects of the insurance scheme on health outcomes remain controversial. This paper assessed the effectiveness of LTCI pilots on health outcomes, using nationally representative longitudinal data of CHARLS. We employed a rigorous causal inference approach to determine the effects of LTCI coverage. LTCI significantly improved self-rated health and cognitive function in older adults. The results were robust when keeping only those living in pilot cities or non-pilot cities as the control group. The effects of LTCI were especially manifested in older adults with physical or intellectual disability.

 The implementation of LTCI resulted in significant improvements in health outcomes for older adults. LTCI improved self-rated health and cognitive function, which were consistent with existing research findings that publicly funded LTC had a positive impact on self-reported health.^7,9,24^ LTCI care beneficiaries had fewer declines in cognitive function^24^ and disability.^12^ A home-based intervention program targeting underlying impairments in physical abilities could reduce the progression of functional decline among physically frail older adults.^32^ However, for physical function, kinds of chronic diseases, and depression, there were no significant improvements. A possible explanation for the mixed evidence could be that the improvements in self-rated health might have been easier to achieve. The LTCI program might not have been in place for a long enough time to produce significant improvements in physical function, chronic diseases, and depression.

 Furthermore, this study showed that older adults with physical or intellectual disability benefited more from LTCI in terms of health outcomes, indicating the rationality of the LTCI eligibility criteria for disabled older adults. LTCI could have significant impacts on health outcomes of older adults with disabilities. Physical and intellectual disabilities can limit an individual’s ability to perform daily activities and negatively impact physical and mental health.^33^ The LTCI program provides much-needed support and care to these individuals, leading to improved self-rated health status and cognitive function. Therefore, policy-makers and healthcare providers should prioritize the needs of older adults with disabilities, ensure that they have access to the LTCI program, and provide appropriate support and care tailored to their specific needs.^34^ It can ensure that resources are used most effectively to improve health outcomes for older adults.

 The positive impacts of the LTCI program on health outcomes among older adults are clear, but the stringent eligibility criteria limit the number of beneficiaries, even in pioneer cities with a broader LTCI coverage.^35^ This suggests that there is a significant unmet demand for LTC services in China, and that the current policy may not meet the needs of the aging population. Additionally, the proportion of older adults receiving benefits from LTCI in China is significantly lower than that in developed countries. Less than 2% of Chinese older adults aged 60 years and over has received benefits from LTCI by the end of 2017,^8^ while in Japan and Germany, LTCI provides benefits to 13.5% and 10.5% of their population aged 65+, respectively.^36^ This disclosed a large gap between the needs of disabled people and access to LTC benefits in China’s LTCI policy experimentation. There is an urgent need to expand the scope and coverage of LTCI in China, to ensure that all eligible older adults have access to the support and care they need.

 The contribution of this paper to the literature is mainly reflected in the following aspects. Firstly, the study provided robust evidence for the positive impacts of the LTCI program on health outcomes among older adults in China, utilizing rigorous DID and PSM methods, to control for confounding factors and identify causal effects. Secondly, a range of important health outcomes were studied among older adults, including self-reported health status, physical function, chronic diseases, cognitive function, and mental health. Thirdly, we explored the health effects of LTCI in 15 pilot cities and two provinces, so as to derive a full picture. Lastly, this paper further examined heterogeneity of health effects across different populations, which provided valuable information for policy-makers and healthcare providers on how to target resources and interventions more effectively to improve health outcomes for the most vulnerable populations. It also justified the LTCI eligibility and called for expanded policies.

###  Limitations of the Study

 This study had several potential limitations. Firstly, due to the lack of individual-level data on the actual use of LTCI in the CHARLS data, we could not derive an exact estimate of the effects of LTCI. Secondly, there was no information on individuals’ eligibility for LTCI benefits. The intent-to-treat effects estimated in this study could include both direct effects for LTCI beneficiaries and spillover effects for nonusers covered by the program, so that the effects of the program for the beneficiaries might be underestimated.^37,38^ Thirdly, due to data limitation, we could not separate the effects of home, community, and institutional care benefits.

## Conclusion

 Overall, based on a quasi-experiment design, this study provides empirical evidence for the effectiveness of LTCI in improving self-rated health status and cognitive function for Chinese older adults, especially those with physical or cognitive disabilities. These findings have important policy implications for expanding LTCI pilots in China and development of LTCI in other middle-income or developing countries, as it could promote the health and well-being of older adults who were most vulnerable. It also highlighted the importance of developing policies that addressed the LTC needs of older adults, so as to secure their health.

## Acknowledgements

 We would like to thank all the participants in the CHARLS.

## Ethical issues

 The ethics application for collecting data on human subjects was approved and updated annually by Peking University’s Institutional Review Board (No. IRB00001052-11015). All participants provided written informed consent.

## Competing interests

 Authors declare that they have no competing interests.

## Supplementary files

Supplementary file 1 contains Text S1, Tables S1-S8, and Figure S1.
