# Supplementary material for: Effects of the Long-term Care Insurance on Health Among Older Adults: A Panel Data From China
Source: Int J Health Policy Manag. 2023 Aug 22;12:7664. doi: 10.34172/ijhpm.2023.7664 (PMC10590242; doi:10.34172/ijhpm.2023.7664)
Supplement: Supplementary file 1 — contains Text S1, Tables S1-S8, and Figure S1. [file ijhpm-12-7664-s001.pdf]

Article title: Effects of the Long-term Care Insurance on Health Among Older Adults: A Panel Data from China

Journal name: International Journal of Health Policy and Management (IJHPM)

Authors' information: Xin Ye[1,2]*, Mingzheng Hu[3,4], Hugo Lin[5]

[1]Institute for Global Public Policy, Fudan University, Shanghai, China.

[2]LSE-Fudan Research Centre for Global Public Policy, Fudan University, Shanghai, China.

[3]School of Public Health, Peking University, Beijing, China.

[4]China Center for Health Development Studies, Peking University, Beijing, China.

[5]CentraleSupélec, Paris-Saclay University, Paris, France.

***Correspondence to**: Xin Ye, Email: yexin@fudan.edu.cn

Citation: Ye X, Hu M, Lin H. Effects of the long-term care insurance on health among older adults: a panel data from China. Int J Health Policy Manag. 2023;12:7664. doi:10.34172/ijhpm.2023.7664

Supplementary file 1

Text S1:
Table S1:
Table S2:
Table S3:
Table S4:
Table S5:
Table S6:
Table S7:
Figure S1:

Text S1. Institutional background for the Long-Term Care (LTC) services

In China, the medical insurance system consists of three basic insurances: the urban employee basic medical insurance scheme (UEBMI), the urban resident basic medical insurance scheme (URBMI), and the new rural cooperative medical system (NRCMS) for rural residents.[1] Through these three pillars, China's medical insurance system covers almost the entire Chinese population.[2] The expansion of health insurance has greatly improved access to health care, especially for older adults in poor health.[3] However, the increasing LTC needs of frail and disabled older people have become a challenge. There is a decline in informal care provided by family members due to smaller family sizes and increased labor mobility. People may even go to hospitals to seek LTC services, resulting in hospital bed congestion and increased medical

expenditures.[4]

To ensure that older people have access to affordable care services, the Chinese government announced in July 2016 the launch of LTCI pilot projects in 15 cities and two provinces (i.e., Jilin and Shandong provinces). Some cities, such as Qingdao and Changchun, had already launched LTCI before the official announcement, whereas Shandong and Jilin provincial governments could select some cities for piloting. The LTCI design varies with economic development, population aging, and fiscal capacities across pilot cities. Supplementary Table 1 summarizes the characteristics of LTCI pilots, including the time of introducing LTCI, the eligibility of the insured, and whether they are included in the study. All the pilots cover urban employees and retirees enrolled in UEBMI, and some also include urban residents enrolled in URBMI, as well as both urban and rural enrollees of URRBMI.[3]

To be eligible for LTCI benefits, individuals must have had a physical or intellectual disability for at least six months, as determined by disability assessments based on the Barthel ADL index or other assessment tools. Most LTCI pilots cover three types of LTC services, namely, home care, institutional care, and hospital care. Home care include home and community social services, such as basic care services (e.g., feeding, bathing, and safety care) and basic medical services (e.g., nursing, rehabilitation, and counseling). Institutional care includes long-term residence and services in designated residential care facilities or nursing homes. Hospital care is provided in LTC beds by designated medical facilities. The type and frequency of LTC services available to beneficiaries depended on the severity of their disability.

The packages of LTCI vary from city to city in terms of expense reimbursement. Some reimburse users with a fixed percentage of the total expenditure, with or without a cap and within a specified period of time. Other cities reimburse a certain amount on a daily or monthly basis and limit the total number off hours or days that can be reimbursed. There are no cash benefits. Most cities pay service providers either by service or by the day. However, there are few regulations on whether and how much a provider can charge the users on top of what LTCI pays the providers, leaving users exposed to uncertain financial risks. In September 2020, the Chinese government expanded the LTCI pilots to 14 additional cities and set out a policy framework to establish a unified LTCI system by 2025.

References

1. Cheng T-M. Early results of China's historic health reforms: the view from Minister Chen Zhu. *Health Affairs.* 2012;31(11):2536-2544.

2. Meng Q, Fang H, Liu X, Yuan B, Xu J. Consolidating the social health insurance schemes in China: towards an equitable and efficient health system. *The Lancet.* 2015;386(10002):1484-1492.

3. Fang H, Eggleston K, Hanson K, Wu M. Enhancing financial protection under China's social health insurance to achieve universal health coverage. *bmj.* 2019;365.

4.Feng J, Wang Z, Yu Y. Does long-term care insurance reduce hospital utilization and medical expenditures? Evidence from China. *Social Science & Medicine.* 2020;258:113081.

Table S1. List of China's LTCI pilot cities

Inclusion	Province	City	Date	Insurance requirements	Eligibility
Yes	Hebei	Chengde	2017-12	UEBMI	Barthel score < 40
Yes	Heilongjiang	Qiqihar	2017-10	UEBMI	Severe disability
Yes	Shanghai	Shanghai	2017-01	UEBMI; URBMI; URRBMI	Aged > 60 years, disability level 2-6 (based on a self-designed scale)
Yes	Jiangsu	Suzhou	2017-06	UEBMI; URBMI; URRBMI	Severe and moderate disability
Yes	Zhejiang	Ningbo	2017-12	UEBMI	Severe disability
Yes	Anhui	Anqing	2017-03	UEBMI	Barthel score < 40
Yes	Jiangxi	Shangrao	2016-11	UEBMI	Severe disability
Yes	Hubei	Jingmen	2016-11	UEBMI; URBMI; URRBMI	Barthel score < 40
Yes	Guangdong	Guangzhou	2017-08	UEBMI	Severe disability, dementia plus moderate disability
Yes	Chongqing	Chongqing	2018-01	UEBMI	Severe disability
Yes	Sichuan	Chengdu	2017-07	UEBMI	Severe disability
Yes	Jilin	Jilin	2016-11	UEBMI; URBMI	Severe disability
Yes	Shandong	Linyi	2017-08	UEBMI	Severe disability
Yes	Shandong	Liaocheng	2017-10	UEBMI	Severe disability
Yes	Shandong	Binzhou	2017-12	UEBMI	Severe disability
No (Not	Jiangsu	Nantong	2016-	UEBMI; URBMI;	Barthel score < 40

in CHARLS)			01	URRBMI	
No (Not in CHARLS)	Xinjiang	Shihezi	2017-01	UEBMI; URBMI; URRBMI	Barthel score < 40
No (Not in CHARLS)	Jilin	Changchun	2015-12	UEBMI; URBMI	Barthel score < 40
No (Not in CHARLS)	Jilin	Tonghua	2017-09	UEBMI; URBMI	Severe disability
No (Not in CHARLS)	Jilin	Songyuan	2016-06	UEBMI; URBMI	Severe disability
No (Not in CHARLS)	Jilin	Meihekou	2017-09	UEBMI; URBMI	Severe disability
No (Not in CHARLS)	Jilin	Hunchun	2017-09	UEBMI; URBMI	Severe disability
No (Not in CHARLS)	Shandong	Zibo	2018-01	UEBMI	Severe disability
No (Not in CHARLS)	Shandong	Dongying	2018-03	URRBMI	Severe disability
No (Not in CHARLS)	Shandong	Jining	2017-12	UEBMI	Severe disability
No (Not in CHARLS)	Shandong	Tai’an	2018-01	UEBMI	Severe disability
No (Not in CHARLS)	Shandong	Rizhao	2018-01	UEBMI	Severe disability

No (Not 2015-2018)	Shandong	Qingdao	2012-07<br>2014-12	UEBMI<br>URBMI; URRBMI	Disability level 3-5 (based on a self-designed scale), dementia
No (Not 2015-2018)	Shandong	Weifang	2014-11	UEBMI	Severe disability
No (Not 2015-2018)	Shandong	Jinan	2018-11	UEBMI	Severe disability
No (Not 2015-2018)	Shandong	Zaozhuang	2018-07	UEBMI	Severe disability
No (Not 2015-2018)	Shandong	Yantai	2018-06	UEBMI	Severe disability
No (Not 2015-2018)	Shandong	Weihai	2018-07	UEBMI	Severe disability
No (Not 2015-2018)	Shandong	Dezhou	2018-12	UEBMI	Severe disability
No (Not 2015-2018)	Shandong	Heze	2018-05	UEBMI	Severe disability

Note: UEBMI, Urban Employee Basic Medical Insurance; URBMI, Urban Resident Basic Medical Insurance; URRBMI, Urban and Rural Resident Basic Medical Insurance.

Table S2. Test for sample attrition bias for the 2015-2018 panel

Dependent Variable	Loss to follow-up in 2018 (N = 4,643)
Treat	-0.14 (0.32)
Age	0.06*** (0.005)
Male	0.37*** (0.06)
Married	-0.35*** (0.07)
Literate	-0.14 (0.08)

Primary school	-0.05 (0.09)
Junior high school and above	-0.05 (0.11)
Urban	0.53*** (0.09)
Number of living children	-0.05* (0.02)

Note: Standard errors are clustered at the city level. The significance levels of 0.1%, 5%, and 1% are denoted by ***, **, and *, respectively.

Table S3. Conversion of ADLs in CHARLS to the measurement of Barthel Index

	ADLs in CHARLS	Barthel Index
**Q1**	**Because of health and memory problems, do you have any difficulty with eating, such as cutting up your food? (Definition: By eating, we mean eating food by oneself when it is ready)**	**Feeding**
R1	1 No, I don't have any difficulty	10 Independent
	2 I have difficulty but can still do it	10 Independent
	3 Yes, I have difficulty and need help	5 Needs help
	4 I can not do it	0 Unable
**Q2**	**Because of health and memory problems, do you have any difficulty with bathing or showering?**	**Bathing**
R2	1 No, I don't have any difficulty	5 Independent
	2 I have difficulty but can still do it	5 Independent
	3 Yes, I have difficulty and need help	0 Unable
	4 I can not do it	0 Unable
**Q3**	**(a) Do you have difficulty with reaching or extending your arms above shoulder level? (he/she is regarded as not having difficulty only if he/she can extend both of his/her arms, otherwise he/she is regarded as having difficulty.)** **(b) Do you have difficulty with picking up a small coin from a table?**	**Grooming**
R3	1 No, I don't have any difficulty	5 Independent
	2 I have difficulty but can still do it	5 Independent
	3 Yes, I have difficulty and need help	0 Unable
	4 I can not do it	0 Unable
**Q4**	**Because of health and memory problems, do you have any difficulty with dressing? Dressing includes taking clothes out from a closet, putting them on, buttoning up, and fastening a belt.**	**Dressing**

R4	1 No, I don't have any difficulty	10 Independent
	2 I have difficulty but can still do it	10 Independent
	3 Yes, I have difficulty and need help	5 Needs help
	4 I can not do it	0 Unable
**Q5**	**Because of health and memory problems, do you have any difficulties with controlling urination and defecation? If you use a catheter (conduit) or a pouch by yourself, then you are not considered to have difficulties.**	**Bowel control**
R5	1 No, I don't have any difficulty	10 Continent
	2 I have difficulty but can still do it	10 Continent
	3 Yes, I have difficulty and need help	5 Occasional accident
	4 I can not do it	0 Independent (or needs to be given enemas)
**Q6**	**Because of health and memory problems, do you have any difficulties with controlling urination and defecation? If you use a catheter (conduit) or a pouch by yourself, then you are not considered to have difficulties.**	**Bladder control**
R6	1 No, I don't have any difficulty	10 Continent
	2 I have difficulty but can still do it	10 Continent
	3 Yes, I have difficulty and need help	5 Occasional accident
	4 I can not do it	0 Independent (catheterized, unable to manage alone)
**Q7**	**Because of health and memory problems, do you have any difficulties with using the toilet, including getting up and down?**	**Toilet use**
R7	1 No, I don't have any difficulty	10 Independent
	2 I have difficulty but can still do it	10 Independent
	3 Yes, I have difficulty and need help	5 Needs help
	4 I can not do it	0 Unable
**Q8**	**Do you have any difficulty with getting into or out of bed?**	**Transfers (bed to chair and back)**
R8	1 No, I don't have any difficulty	15 Independent
	2 I have difficulty but can still do it	10 Needs minor help (verbal or physical)
	3 Yes, I have difficulty and need help	5 Needs major help (1-2 people, physical), can sit
	4 I can not do it	0 Unable

**Q9**	**Do you have difficulty with walking 100 meters?**	**Mobility on level surfaces**
R9	1 No, I don't have any difficulty	15 Independent (but may use any aid, e.g. stick) >50 yards
	2 I have difficulty but can still do it	10 Walks with help of one person (verbal or physical) >50 yards
	3 Yes, I have difficulty and need help	5 Wheelchair independent, including corners, >50 yards
	4 I can not do it	0 Immobile or <50 yards
**Q10**	**Do you have difficulty with climbing several flights of stairs without resting?**	**Stairs**
R10	1 No, I don't have any difficulty	10 Independent
	2 I have difficulty but can still do it	10 Independent
	3 Yes, I have difficulty and need help	5 Needs help (verbal, physical, carrying aid)
	4 I can not do it	0 Unable

Table S4. Parallel trend tests using CHARLS 2011, 2013, 2015 and 2018

Dependent variables	DID with matching (1)	Pre-trend test (2)		
	Coefficient on Treat × 2018 (post)	Coefficient on Treat × 2011 (pre)	Coefficient on Treat × 2013 (pre)	Coefficient on Treat × 2018 (post)
Control group: Not covered by LTCI				
Self-rated health status	0.14* (0.06)	0.13 (0.09)	0.16 (0.11)	0.15* (0.02)
Physical function	1.87 (3.43)	1.40 (8.93)	1.79 (3.32)	1.81 (3.55)
Kinds of chronic diseases	-0.55 (0.85)	-0.31 (0.62)	-0.45 (0.39)	-0.42 (0.53)
Cognitive function	0.57** (0.18)	0.55 (0.40)	0.56 (0.88)	0.58** (0.20)
Depression	-0.54 (0.59)	-0.31 (0.27)	-0.45 (0.83)	-0.41 (1.91)
Control group: Not covered by LTCI in pilot cities				
Self-rated health status	0.20* (0.09)	0.10 (0.06)	0.14 (0.10)	0.13* (0.06)
Physical function	6.26 (5.16)	1.43 (1.72)	1.60 (3.17)	2.57 (1.65)
Kinds of chronic	-0.49*** (0.04)	-0.29 (0.18)	-0.25 (0.43)	-0.34 (0.40)

diseases				
Cognitive function	0.71*** (0.16)	0.35 (0.21)	0.74 (2.30)	0.45*** (0.05)
Depression	-1.25*** (0.05)	-0.36 (0.38)	-0.36 (0.19)	-0.24 (0.13)
Control group: Not covered by LTCI in non-pilot cities				
Self-rated health status	0.18** (0.06)	0.28 (0.25)	0.12 (0.07)	0.12*** (0.04)
Physical function	2.81 (3.42)	1.28 (1.73)	2.78 (1.65)	2.55 (1.65)
Kinds of chronic diseases	-0.29 (0.18)	-0.24 (0.36)	-0.35 (0.21)	-0.48 (0.31)
Cognitive function	0.53*** (0.01)	0.35 (0.21)	0.79 (0.45)	0.44** (0.15)
Depression	-0.79 (0.45)	-0.30 (0.48)	-0.34 (0.48)	-0.31 (0.49)

Note: Standard errors are clustered at the city level. The significance levels of 0.1%, 5%, and 1% are denoted by ***, **, and *, respectively. In column 2, we run a specification that includes three interaction terms, Treat × 2011, Treat × 2013, and Treat × 2018, with wave 2015 as the reference. All regressions control for individual fixed effects, year fixed effects, and individual covariates.

Table S5. The distribution of treated and control groups in the spillover study

	**Wave 2015 (n = 9,040)**			**Wave 2018 (n = 9,040)**		
	**Treated (n=2,122)**	**Control (n=6,918)**	**p**	**Treated (n=2,122)**	**Control (n=6,918)**	**p**
Outcome variables						
Self-rated health status (0-2)	1.05 (0.02)	0.96 (0.01)	<0.001	0.97 (0.02)	0.85 (0.01)	<0.001
Physical function (0-100)	65.16 (0.71)	68.15 (0.37)	<0.001	67.19 (0.67)	70.10 (0.34)	<0.001
Kinds of chronic diseases (0-12)	2.12 (0.04)	2.33 (0.02)	<0.001	0.68 (0.02)	0.79 (0.01)	<0.001
Cognitive function (0-21)	9.57 (0.10)	9.00 (0.06)	<0.001	8.57 (0.10)	8.22 (0.06)	0.004
Depression (0-30)	7.36 (0.14)	8.58 (0.08)	<0.001	6.91 (0.14)	7.97 (0.08)	<0.001
Covariates						
Age	68.19 (0.15)	68.04 (0.08)	0.38	71.19 (0.15)	71.04 (0.08)	0.38
Sex			0.42			0.42
Male	1,040 (49.01%)	3,321 (48.01%)		1,040 (49.01%)	3,321 (48.01%)	
Female	1,082 (50.99%)	3,597 (51.99%)		1,082 (50.99%)	3,597 (51.99%)	
Marital status			0.028			0.062
Single	364 (18.13%)	1,334 (20.37%)		492 (23.19%)	1,742 (25.18%)	
Married	1,644 (81.87%)	5,215 (79.63%)		1,630 (76.81%)	5,176 (74.82%)	
Education level			<0.001			<0.001
Illiterate	626 (29.50%)	2,455 (35.49%)		626 (29.50%)	2,455 (35.49%)	

Literate	473 (22.29%)	1,372 (19.83%)		473 (22.29%)	1,372 (19.83%)	
Primary school	564 (26.58%)	1,609 (23.26%)		564 (26.58%)	1,609 (23.26%)	
Junior high school and above	459 (21.63%)	1,482 (21.342%)		459 (21.63%)	1,482 (21.342%)	
Residence			<0.001			<0.001
Urban	518 (26.88%)	1,319 (21.46%)		534 (27.65%)	1,352 (21.97%)	
Rural	1,409 (73.12%)	4,826 (78.54%)		1,397 (72.35%)	4,801 (78.03%)	
Smoking			0.143			0.096
Never	1,058 (52.87%)	3,576 (54.74%)		1,128 (53.49%)	3,830 (55.55%)	
Yes or ever	943 (47.13%)	2,957 (45.26%)		981 (46.51%)	3,065 (44.45%)	
Drinking			0.898			0.472
Never	1,077 (53.90%)	3,506 (53.74%)		1,132 (53.65%)	3,638 (52.76%)	
Yes or ever	921 (46.10%)	3,018 (46.26%)		978 (46.35%)	3,258 (47.24%)	
Number of living children	3.00 (0.03)	3.29 (0.02)	<0.001	2.84 (0.03)	3.12 (0.02)	<0.001

Note: Standard deviations are in parentheses; standard errors clustered at the city level are in brackets in the last column.

Table S6. Spillover effects of LTCI on health outcomes

Dependent variables	Coefficient on Treat × Post	
	**DID (1)**	**DID with matching (2)**
Self-rated health status	0.03 (0.02)	0.03 (0.02)
Physical function	0.31 (0.94)	0.45 (0.90)
Kinds of chronic diseases	-0.11 (0.10)	-0.14 (0.11)
Cognitive function	0.23 (0.18)	0.27 (0.19)
Depression	-0.08 (0.23)	-0.05 (0.22)
Year FE	Y	Y
Individual FE	Y	Y

Note: Standard errors are clustered at the city level. The significance levels of 0.1%, 5%, and 1% are denoted by ***, **, and *, respectively. All regressions control for year FE, individual FE, and individual covariates.

Table S7. Heterogeneous effects of LTCI by physical function

	LTCI × time × physical function	
	DID	DID with matching
	(1)	(2)
Self-rated health status	0.27	0.20
	(0.30)	(0.36)
Kinds of chronic	-0.43	-1.16

diseases		
	(0.71)	(1.23)
Cognitive function	1.40	2.75**
	(1.02)	(1.04)
Depression	-2.11	-3.95
	(1.80)	(2.16)

Note: Standard errors are clustered at the city level. The significance levels of 0.1%, 5%, and 1% are denoted by ***, **, and *, respectively. All regressions control for individual fixed effects, year fixed effects, and individual covariates.

Table S8. Heterogeneous effects of LTCI by intellectual function

	LTCI × time ×intellectual function	
	DID	DID with matching
	(1)	(2)
Self-rated health status	-0.16	-0.24*
	(0.09)	(0.11)
Physical function	7.56	10.51*
	(5.31)	(4.93)
Kinds of chronic diseases	0.45	0.72
	(0.30)	(0.41)
Depression	-1.08	0.58
	(1.11)	(1.54)

Note: Standard errors are clustered at the city level. The significance levels of 0.1%, 5%, and 1% are denoted by ***, **, and *, respectively. All regressions control for individual fixed effects, year fixed effects, and individual covariates.

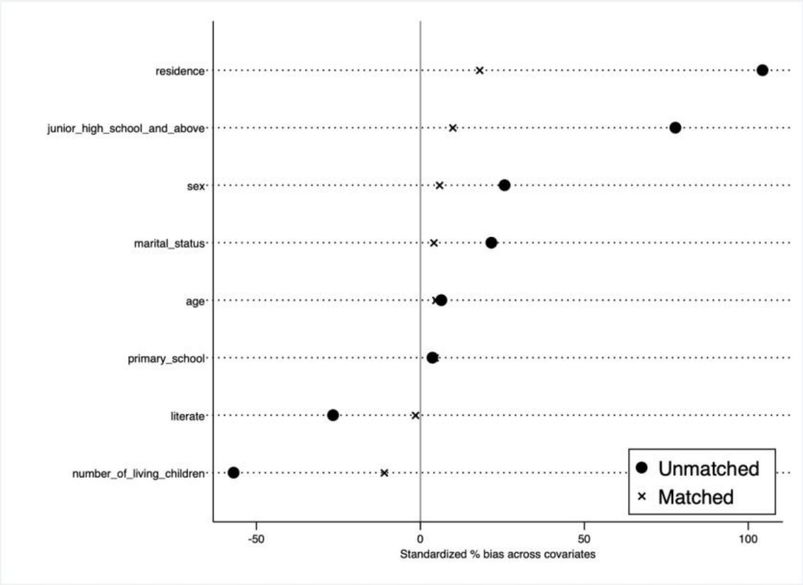

Figure S1. Distribution of propensity scores before and after matching
